# Industry Payments Received by Residents During Training

**DOI:** 10.1001/jamanetworkopen.2023.37904

**Published:** 2023-10-16

**Authors:** Sean O. Hogan, Kenji Yamazaki, Yuezhou Jing, Bruce J. Trock, Misop Han, Eric Holmboe

**Affiliations:** 1 Accreditation Council for Graduate Medical Education, Chicago, Illinois; 2 Johns Hopkins University School of Medicine, Baltimore, Maryland

## Abstract

**Importance:**

Those responsible for medical education—specialties, sponsoring institutions, and program directors (PD)—are independently associated with the professional identity formation of the trainees with respect to potential conflicts of interest.

**Objective:**

To identify the relative degree to which factors in the training environment are associated with resident acceptance of payments from pharmaceutical and medical device companies.

**Design, Setting, and Participants:**

Cross-sectional, retrospective study of residents enrolled in the 3 largest primary-care specialties (internal medicine [IM], family medicine [FM], obstetrics and gynecology [OBGYN]) and 3 largest surgical disciplines (general surgery [GS], orthopedic surgery, and urology) during academic year 2020 to 2021. All analyses were conducted January through August 2023.

**Exposures:**

Specialty, sponsoring institutions’ ownership (nonprofit, for-profit, federal government, local government, or state government), and the number of payments PDs accepted.

**Main outcomes and measures:**

Modified Poisson regression assessed the relative risk of ownership, specialty, and PD behavior on residents’ acceptance of industry payments as recorded in the Open Payments Program (OPP) database.

**Results:**

In total, there were 124 715 residents in all training programs during 2020 to 2021, 12% of whom received payments totaling $6.4 million. There were 65 992 residents in training during 2020 to 2021 in the 6 specialties evaluated in this study, with 4438 in orthopedics, 1779 in urology, 9177 in GS, 5819 in OBGYN, 14 493 in FM, and 30 286 in IM. OPP records $3.9 million in payments to the 8750 residents (13.4%) who received at least 1 industry payment. The record of all payments to residents in OPP totals $6.4 million. Compared with residents in federal sponsoring institutions, those affiliated with for-profit institutions were 3.50 (95% CI, 2.32-5.28) times more likely to accept industry payments, while those affiliated with nonprofit organizations were 2.00 (95% CI, 1.36-2.93) times more likely to accept payments. Compared with IM, residents in each of the following specialties have an elevated risk of accepting payments: orthopedics, 3.21 (95% CI, 2.73-3.77) times; urology, 2.95 (95% CI, 2.44-3.56) times; GS, 1.21 (95% CI, 1.00-1.45) times; OBGYN, 1.30 (95% CI, 1.05-1.62) times. The difference in the risk of accepting a payment between FM and IM residents was not statistically significant. The number of payments PDs accepted slightly elevated the risk of residents to accept a payment by 1.01 (95% CI, 1.01-1.01).

**Conclusions and relevance:**

In this cross-sectional, retrospective study, receipt of industry payments by residents was associated with specialty, institutional control, and PD behavior.

## Key Points

**Question:**

To what degree are various factors of the medical learning environment associated with the probability of learners’ acceptance of payments from pharmaceutical and medical device manufacturers?

**Findings:**

In a cross-sectional study of 65 992 residents in the 3 largest primary-care specialties and 3 largest surgical disciplines, physicians in privately owned sponsoring institutions had an elevated probability of accepting industry payments. Compared with residents in internal medicine, residents in all other specialties had an elevated probability of having received an industry payment.

**Meaning:**

These findings suggest that financial relationships between physicians and the pharmaceutical and medical device industries may form during medical training, and the probability for trainees to accept industry payments is associated with the type of sponsoring institution and the specialty.

## Teaser

This cross-sectional study evaluates the degree to which various factors of the medical learning environment are associated with the probability of learners accepting payments from pharmaceutical and medical device manufacturers.

## Introduction

Professional identity formation is a crucial component of residency training.^1^ The National Academy of Medicine (NAM, formerly the Institute of Medicine) recognized that medical residents’ ethical formation is susceptible to the influence of their mentors, especially in cases of potential conflicts of interest. Although they acknowledge the importance of industry-practitioner relationships that usher in valuable scientific advancements, the NAM expressed concern that “financial ties to industry may unduly influence professional judgments involving the primary interests and goals of medicine.”^2^

The NAM’s concerns are more than conjecture. The pharmaceutical industry spent nearly US $30 billion on marketing in 2016, two-thirds of which was direct to health care practitioners.^3^ Industry payments to physicians are correlated with higher tendencies for opiate prescribing^4,5^ and writing for name-brand drugs over less-expensive generics.^6,7,8^ Industry interactions with trainees are associated with a lower inclination of residents to rely on evidence-based prescribing choices.^9^ As far as we know, no peer-reviewed publication examining industry payments to physicians has failed to find an association between payments and professional decisions working to the benefactor’s advantage.^10^ In fact, DeJong and colleagues^6^ found payments, such as meals worth as little as $20, are correlated with prescription behaviors favoring the entities who make the payments.

During medical training, much of the hidden curriculum that residents learn comes by intuiting what is right from wrong by observing others in the learning environment.^11,12^ The tendencies of mentors to accept industry payments is correlated with the propensity of fellows in internal medicine (IM) subspecialty training to follow suit.^13^ The presence of device and drug industry influence in medical education is neither a secret, nor a recent revelation.^14,15^ Survey research has addressed the frequency of contact between industry and learners and resulting attitudes about interactions.^16,17^ But we know of no study that has used the federal Open Payments Program (OPP) data set in an attempt to examine the factors associated with residents accepting industry payments during training.

We examined the patterns of industry payments accepted by residents using the OPP database, which is housed by the Centers for Medicare and Medicaid Services. Federal law requires industry representatives to document their payments to health care professionals. We aim to identify whether this data set can shed light on industry-learner relationships and reveal factors in the learning environment that may be associated with residents’ receipt habits. Specifically, we hypothesized that payment receipt would vary according to specialty, the type of the sponsoring institution, and program director (PD) payment receipt behavior.

The OPP database, established under the Patient Protection and Affordable Care Act of 2013, is intended to provide a comprehensive record of payments made by pharmaceutical and medical device companies to health care practitioners. The OPP database includes information on the recipient’s name and profession (ie, physicians, chiropractors, dentists, and so forth), organizational affiliation, the entity making the payment, the nature and timing of the payment, and its market value.

Payments are categorized in the OPP database as general, research, and ownership. Research payments refer to consideration for facilitating a clinical study. Ownership payments include dividends paid for ownership in a business. This study focuses on general payments made to residents and PDs. General payments include any transfers such as gifts, honoraria, consulting fees, meals, travel, entertainment, or fee waivers.

Although OPP requires reporting of payments to most health professionals, including fellows and independently practicing physicians,^18,13^ residents are an exception. Although the law does not mandate reporting of payments to residents, it does not prohibit it. During our study period, July 1, 2020, until June 30, 2021, 124 715 residents (excluding fellows) were training in Accreditation Council for Graduate Medical Education (ACGME)–accredited programs. Twelve percent (14 817) of them appear in the OPP database as having received a combined $6.38 million in industry payments. Since regulations do not require reporting of payments to residents, these figures are likely an underestimation. Despite these limitations, our hypothesis is that by using the OPP database, we can identify patterns of payments to residents that align with their specialty, gender, PD behavior, and sponsoring institutions.

## Methods

### Participants

This study had a cross-sectional, retrospective design. The acceptance of general payments to residents was analyzed and compared by the type of the sponsoring institution, specialty, resident’s gender, and PD’s payment acceptance. The institutional review board at the American Institute for Research exempted this study from review. Because the OPP data are publicly available, participant consent is not required. This study followed Strengthening the Reporting of Observational Studies in Epidemiology (STROBE) reporting guidelines for cross-sectional studies.

We focused on 65 992 residents in the 6 large specialties who were training in an ACGME-accredited program during the academic year 2020 to 2021. These residents accounted for 52.9% of all residents in training and represented the 3 largest primary-care specialties: IM, obstetrics and gynecology (OBGYN), and family medicine (FM); and the 3 largest surgical specialties: general surgery (GS), orthopedic surgery (OS), and urology. To link residents with their OPP records, we used the national provider identification (NPI) number. Seven residents switched programs during the study period, and we aligned them with the programs they belonged to for the longest term.

PDs were identified through ACGME records, which included their NPIs and their starting or ending dates in that role. We found 168 programs that had more than 1 individual serving as PD during our time frame. We attributed payments to the program that the PD was affiliated with at the time of receipt. For example, a PD could have been employed with 1 program between July and December and received payments then. The same individual could have moved to another organization in the subsequent January through June and accepted no payments at the new program. By using the date of payment, we could attach the association of the PD’s acceptance to the program in which the PD was employed in the July to December time frame. When any program switched PDs, the sum of industry payments to the program was treated as the number of receptions for the PD of the individual program, regardless of who the individual person was.

We used data from the ACGME to locate information about the residents’ training programs, including the specialty and the type of sponsoring institution (for-profit, nonprofit, local, state, or federal government agencies). We categorized residents’ gender as men, women, and other, which encompasses not reported, nonbinary, or another gender identity.

Resident, PD, and sponsoring institution characteristics (such as ownership) were provided by the ACGME. Data about receipt of industry payment came from the OPP. Resident gender was provided by the Association of American Medical Colleges. PDs and residents are matched to the OPP data by use of NPI number.

The outcome variable is the receipt of general payments during the 2020 to 2021 academic year. We did not include research and ownership payments. Residents who appeared in the general payments database were coded as 1, and those who did not were coded as 0. We kept a continuous count of the number of payments received by residents and PDs and the dollar value of each transfer.

### Statistical Analysis

Modified Poisson regression was used to examine each variable’s association with the residents’ acceptance of a payment according to a generalized estimating equation (GEE) model.^19,20^ Residents were nested within each program, and programs were embedded within each cross-classified cluster by specialties and sponsoring institutions. This data clustering was accounted for in the GEE model, using an exchangeable covariance matrix. The matrix assumes that associations of outcomes are equal in size among peer residents within clusters but are specified as independent between clusters.^21^

In the regression model, we measure the increased risk of residents in 1 group to receive a payment over those in a reference group. We chose as a reference the group that received the fewest industry payments. For specialty, that was IM. For gender, women received the least. For sponsoring institution type, it was federally owned. Results are reported as relative risk and 95% CIs for ownership type of sponsoring institution, medical specialty, resident gender, and PD receipt. Two-tailed *P* values were considered statistically significant at *P* < .05. All statistical analyses were performed using SAS Enterprise Guide, version 7.15 (SAS Institute).

## Results

There were 65 992 residents training in accredited programs in the 6 specialties included in this study during the 2020 to 2021 academic year. Of them, 30 749 identified as women (46.6%), 34 258 identified as men (51.9%), and the balance, 985, another gender identity or declined to answer (1.5%). IM had the largest number of residents (30 286). The next largest specialty was FM, followed by GS, OBGYN, and then OS. FM had the largest number of programs with 678. Most programs were situated in private, nonprofit sponsoring institutions (1464 of 2182 programs [67.1%]); followed by state systems; then private, for-profit organizations; the federal government; and local governments. Table 1 summarizes the characteristics of residents, specialties, and sponsoring institutions. Overall, 14 817 residents were reported to have received nearly $6.4 million in a single academic year, and 8750 residents among the 6 specialties in the analysis received $3.9 million.

**Table 1.** Characteristics of Residents, Programs, and Sponsoring Institutions for 6 Specialties

Residents	Participants, No. (%)						
	OS (n = 4438)	Urology (n = 1779)	GS (n = 9177)	OBGYN (n = 5819)	FM (n = 14 493)	IM (n = 30 286)	Total (N = 65 992)
Gender^a^							
Men	3679 (82.9)	1272 (71.5)	5098 (55.6)	864 (14.8)	6583 (45.4)	16 762 (55.3)	34 258 (51.9)
Women	745 (16.8)	502 (28.2)	4022 (43.8)	4918 (84.5)	7730 (53.3)	12 832 (42.4)	30 749 (46.6)
Other	14 (0.3)	5 (0.3)	57 (0.6)	37 (0.6)	180 (1.2)	692 (2.3)	985 (1.5)
Programs							
Total No.	199	145	328	284	678	548	2182
Residents in training, median (IQR)	21.0 (15.0-27.0)	12.0 (10.0-15.0)	24.0 (17.0-36.0)	18.0 (15.0-24.0)	20.0 (15.0-26.0)	42.0 (29.0-70.0)	23.0 (16.0-35.0)
Sponsoring institution							
Federal government	8 (4)	5 (3.4)	9 (2.7)	7 (2.5)	14 (2.1)	10 (1.8)	53 (2.4)
Local government	3 (1.5)	2 (1.4)	9 (2.7)	12 (4.2)	18 (2.7)	19 (3.5)	63 (2.9)
Private, for-profit	7 (3.5)	2 (1.4)	27 (8.2)	10 (3.5)	53 (7.8)	63 (11.5)	162 (7.4)
Private, nonprofit	125 (62.8)	92 (63.4)	214 (65.2)	188 (66.2)	476 (70.2)	369 (67.3)	1464 (67.1)
State government	56 (28.1)	44 (30.3)	69 (21)	67 (23.6)	117 (17.3)	87 (15.9)	440 (20.2)

Abbreviations: FM, family medicine; GS, general surgery; IM, internal medicine; OBGYN, obstetrics and gynecology; OS, orthopedic surgery.

^a^ Gender identification is based on data provided by the Association of American Medical Colleges (AAMC).

### Payment Amounts

A total of 26 611 payments were made to residents in 2020 to 2021. Of the 65 992 residents, 616 residents were excluded because their NPIs were missing or duplicate. The 7 residents who changed programs were analyzed using data for the program they were enrolled in for the greatest period of time, resulting in 26 526 separate payments used for analysis. Table 2 describes payments received by residents and PDs. Of the 65 376 residents training during the study period, 8750 (13.4%) reportedly accepted at least 1 industry payment. Of the 2466 individuals who served as PD, 976 (39.6%) had accepted industry payments worth a total of $5.2 million. OS residents and PDs received the most total payments at $2.2 million and $4 million, respectively. OBGYN residents and PDs received the lowest total payments at $68 110 and $185 817, respectively.

**Table 2.** Industry Payments Received by Residents and PDs

Residents	No. (%)						
	OS (n = 4382)	Urology (n = 1776)	GS (n = 9092)	OBGYN (n = 5774)	FM (n = 14 362)	IM (n = 29 990)	Total (N = 65 376)^a^
Residents with payments	1709 (39.0)	628 (35.4)	1171 (12.9)	734 (12.7)	1817 (12.7)	2691 (9.0)	8750 (13.4)
Residents receiving in total >$1000, No. (% of total [% of recipients])	691 (15.8 [40.4])	28 (1.6 [4.5])	48 (0.5 [4.1])	2 (<0.5 [<0.5])	5 (<0.5 [<0.5])	6 (<0.5 [<0.5])	780 (1.2 [8.9])
Total value of industry payments to residents, $	2 236 741.70	910 218.10	311 374.32	68 110.34	155 390.75	288 588.09	3 970 423.30
Per-resident gift for those who received, median (IQR), $	526.32 (131.20-1400.75)	115.06 (43.27-225.33)	81.33 (24.49-190.77)	33.34 (17.68-103.16)	32.50 (16.78-80.58)	39.61 (18.52-117.09)	64.40 (21.04-193.68)
Items as payment, No.	7860	2230	3175	1223	4648	7390	26 526
Value of each payment to residents who received, median (IQR) [range], $	112.75 (68.61-274.37) [0.91-20 000.00]	44.17 (26.91-69.05) [5.03-50 000.00]	34.06 (18.31-87.78) [2.45-25 000.00]	22.05 (17.26-54.83) [7.86-5200.00]	17.06 (15.22-22.34) [6.16-10 000.00]	19.36 (16.00-27.55) [0.79-46 874.86]	31.27 (17.47-89.42) [0.79-50 000.00]
PDs, No.	213	154	369	320	775	635	2466^b^
PDs with industry payments	153 (71.8)	96 (62.3)	196 (53.1)	131 (40.9)	219 (28.3)	181 (28.5)	976 (39.6)
PDs receiving payments in total >$1000, No. (% of total) [% of recipients]	75 (35.2) [49.0]	18 (11.7) [18.8]	46 (12.5) [23.5]	23 (7.2) [17.6]	27 (3.5) [12.3]	21 (3.3) [11.6]	211 (8.5) [21.5]
Total value of payments to PDs, $	4 116 497.44	179 574.42	363 608.67	185 816.66	119 710.49	260 838.54	5 226 046.22
Per-PD gift for those who received, median (IQR), $	980.48 (151.62-4060.27)	141.27 (39.08-839.2)	127.21 (44.93-889.93)	91.5 (30.63-300.42)	62.08 (19.85-240.85)	96.83 (25.41-348.3)	119.55 (35.76-614.21)
Items as payment, No.	1794	896	1032	727	2611	2642	9702
Value of each payment to PDs who received, median (IQR) [range], $	69.00 (19.13-450.94) [0.17-41 8127.10]	18.54 (14.21-43.24) [0.28-10 575.00]	24.99 (15.72-93.29) [0.45-11 000.00]	19.85 (14.84-61.02) [0.56-4000.00]	14.93 (12.62-18.48) [0.33-3700.00]	15.89 (12.78-21.01) [0.24-6930.00]	17.68 (13.55-33.68) [0.17-418 127.1]
Programs whose PDs received industry payment	149 (74.9)	95 (65.5)	195 (59.5)	130 (45.8)	219 (32.3)	178 (32.5)	966 (44.3)
Total value of payments to specialty programs, $	4 116 497.44	179 574.42	363 608.67	185 816.66	119 710.49	260 838.54	5 226 046.22
Per-program PD payments, median (IQR) [range], $	1000.00 (162.82-4717.00) [13.11-1 921 199.12]	138.95 (38.32-845.65) [3.82-83 506.42]	129.48 (46.13-899.96) [5.88-24 984.95]	93.01 (30.19-267.87) [11.43-72 600.89]	62.44 (18.99-255.50) [9.54-16 688.12]	96.87 (25.07-368.47) [11.44-54 843.31]	123.92 (35.30-654.46) [3.82-1 921 199.12]

Abbreviations: FM, family medicine; GS, general surgery; IM, internal medicine; OBGYN, obstetrics and gynecology; OS, orthopedic surgery; PD, program director.

^a^ Out of the 65 992 residents, 616 had missing or duplicate NPIs. Thus, 65 376 residents were used for reporting.

^b^ Out of the 2514 PDs, 48 had missing or duplicate NPIs. Thus, 2466 PDs were considered for reporting.

The median (IQR) value of all payments received by residents was $64.40 ($21.04-$193.68). The median (IQR) value of any particular payment was $31.27 ($17.47-$89.42) with a maximum value of $50 000 and a minimum of $0.79. Consistent with previous findings of early career graduates in clinical practice, OS had the highest proportion (39.0%) who accepted payments, and IM had the smallest (9.0%). Of those residents in the OPP database, the median (IQR) total payment to the recipients was highest for OS ($526.32 [$131.20-$1400.75]) and lowest for FM ($32.50 [$16.78-$80.58]).

The median (IQR) value of payments to PDs was $119.55 ($35.76-$614.21). The median (IQR) value of any particular payment was $17.68 ($13.55-$33.68) with a maximum value of $418 127.10 and a minimum of $0.17. OS had the highest proportion (153 of 213 participants [71.8%]) who accepted industry payments, and FM had the smallest (219 of 775 participants [28.3%]). Of those PDs in the OPP database, the median (IQR) total payment to the recipients was highest for OS ($980.48 [$151.62-$4060.27]) and lowest for FM ($62.08 [$19.85-$240.85]). For programs whose directors accepted payments, the maximum was $1.9 million (OS), and the minimum was $3.82 (urology).

### Relative Risk of Accepting Payments

Sponsoring institution control was significantly associated with the probability of a resident to appear in the OPP data set. Table 3 reports the regression analysis indicating that that the relative risk for trainees with for–profit sponsoring institutions was 3.50 times higher than those in federal sponsoring institutions to accept industry payments (95% CI, 2.32-5.28; *P* < .001). Residents affiliated with nonprofit sponsoring institutions were 2.00 times more likely than those in federal sponsoring institutions to have accepted payments (95% CI, 1.36-2.93; *P* < .001). The difference between the probability of residents training in federal sponsoring institutions and those in programs operated by state or local government was not statistically significant.

**Table 3.** Relative Risk for Residents Receiving Industry Payments

Variable	Relative risk (95% CI)	*P* value
Type of sponsoring institution		
For-profit	3.50 (2.32-5.28)	<.001
Nonprofit	2.00 (1.36-2.93)	<.001
Government, local	1.48 (0.87-2.52)	.15
Government, state	1.45 (0.98-2.14)	.07
Government, federal	1 [Reference]	NA
Medical specialty		
Orthopedic surgery	3.21 (2.73-3.77)	<.001
Urology	2.95 (2.44-3.56)	<.001
General surgery	1.21 (1.00-1.45)	.05
Obstetrics and gynecology	1.30 (1.05-1.62)	.02
Family medicine	1.15 (0.97-1.36)	.12
Internal medicine	1 [Reference]	NA
Resident gender^a^		
Men	1.09 (1.06-1.13)	<.001
Women	1 [Reference]	NA
Other^b^	0.85 (0.73-0.99)	.04
PD receipt of payment	1.01 (1.01-1.01)	<.001

Abbreviations: NA, not applicable; PD, program director.

^a^ Gender identification was based on data provided by the Association of American Medical Colleges (AAMC).

^b^ Refers to individuals who are nonbinary, declined to identify, or gave no information.

Specialty was significantly associated with the risk of residents’ acceptance of industry payments. The relative risk of OS residents accepting industry payments was 3.21 times higher than IM residents (95% CI, 2.73-3.77; *P* < .001). Urology residents were 2.95 times more likely than IM residents to accept industry payments (95% CI, 2.44-3.56; *P* < .001). OBGYN residents were 1.30 times more likely (95% CI, 1.05-1.62). GS residents were 1.21 times more likely to accept industry payments (95% CI, 1.00-1.45). FM was 1.15 times more likely to accept industry payments (95% CI, 0.97-1.36). The difference in the risk of accepting a payment between FM and IM residents was not statistically significant.

We found that the probability of men to accept payments was 9% higher than women, whereas those who did not identify a gender, whose data were missing, or who were nonbinary (others) were 15% lower than women. The number of the PD’s receipt of payments had a small but significant association for residents in that training program to accept payments. The number of payments PDs accepted slightly elevated the risk of residents to accept a payment by 1.01 (95% CI, 1.01-1.01).

### Types of Payment

The OPP records broad categories for the nature of these payments (see eTable 1 in Supplement 1 for a count of the types of payments received by residents in the 6 specialties in our study and eTable 2 in Supplement 1 for all specialties). eTable 3 in Supplement 1 defines the OPP categories. Among those categories, refreshments were the largest type of payment received. Nine in 10 payments (23 864 payments [89.96%]) were for food and drink. This was followed by education (2050 payments [7.7%]) and travel and lodging (528 payments [2.0%]). Group meals accounted for 15 822 payments (59.6%), with a total dollar value of $579 259.41. We inferred group meals by identifying records for food and beverages made on the same dates for residents in the same program with identical dollar values.

Table 2 reports that several residents received general payments as high as $50 000 in a single year. The largest individual payments were in the form of grants. Two urology residents each received $50 000 grants, while 1 IM resident received a grant of $46 875. One GS resident received a $25 000 grant. Each of 5 OS residents accepted $20 000 grants. One FM resident received a $10 000 grant. One OBGYN resident accepted a $5200 consulting fee.

## Discussion

About 12% of all residents were reported to have received nearly $6.4 million in a single academic year, despite the fact that such reporting is optional. Although there were instances of residents accepting as much as $50 000 in industry payments in a single year, the vast majority of payments were for refreshments, and many of those were in group settings. Bearing in mind that the typical year-1 resident is carrying $200 000 or more in student debt on a salary of about $60 000,^22^ one may find it difficult to criticize a resident for participating in a sponsored lunch. It is not a state of impecunity alone that may attract the resident to a free lunch. Our data suggest that their colleagues and faculty are attending the same events, thus creating a social environment that condones acceptance of industry payments. The influence of such payments has been associated with prescribing behaviors that benefit the giving pharmaceutical firm.^4,5,6,7,8,10^

These findings highlight the importance of revising the federal exception for trainees in OPP. In 2013, the Centers for Medicare and Medicaid Services concluded that extending the OPP regulations to residents would lead to uneven reporting because too few had an NPI and that state licensure for residents was variable.^23^ Today, the ACGME has NPIs for 99.1% of active residents; thus, the rationale for excluding them no longer holds true. Including residents would provide a more comprehensive understanding of the extent of industry payments in the health care sector, their potential influence on patient care, and the patterns of formation of industry-physician financial relationships during medical education. Given the long-lasting effects of residency training,^24^ specialty societies, medical educators, and sponsoring institutions may elect to reevaluate their policies on conflicts of interest.

Our study did not explore the effects of industry payments on residents’ decision-making. However, they suggest that there is variation in reporting by specialty, sponsoring institution control, and PD behavior. The consequences of these factors of the training environment support the concerns raised by the NAM regarding the potential outcomes of industry payments on the ethical formation of physicians during their training. Although the effect size was small in this study, the association between PDs and resident behavior is present and warrants further investigation. It is possible that the reporting exception for residents may obscure the true effect of the PDs.

We found specialty was associated with payment patterns, with the association most pronounced in OS. This is consistent with other cross-specialty studies of the OPP data set, which find proceduralists,^25^ particularly orthopedic surgeons, have a higher tendency to receive payments. Residents in programs affiliated with privately controlled sponsoring institutions (for-profit and nonprofit) had a higher likelihood of accepting industry payments than those in government-controlled institutions. Although residents in programs affiliated with state and local health systems seem to have appeared in the OPP more than those in federal sponsoring institutions, the difference is not statistically significant. It remains to be determined why this is. State-level gifting policies vary widely, but states with stringent drug detailing regulations see slower uptake of new, expensive drugs.^26,27^

### Limitations

This study had limitations. The federal government does not require reporting of industry payments to residents; thus, we suspect there is coverage error in the OPP. This may result in understating the extent to which transfers are made to residents in certain specialties, programs, or sponsoring institutions. With more complete coverage it is possible that there would be a stronger relative risk associated with gender, as our findings were somewhat lower than those found in studies of industry payments to independently practicing physicians.^18,27^ The minimal relative risk of resident receipt given PD behavior might also come more in line with findings in studies of fellowship programs^13^ had the OPP systematically collected resident payments. Our findings, though, concur with existing literature that suggests procedural specialists, orthopedics in particular, receive more industry payments than primary-care practitioners. Our study spanned only 1 academic year, and resident interactions with industry representatives may have been diminished by the COVID-19 outbreak as industry representatives’ in-person access to medical facilities was curtailed.^28^

## Conclusions

In this cross-sectional study of industry payments to residents, approximately 12% of residents were reported to have accepted industry payments in an academic year. That proportion could be an understatement because the available reporting is strictly voluntary. Specialty, PD behavior, and sponsoring institution control were significantly associated with residents accepting industry payments. Sponsored group meals appear to account for a large proportion of payments, suggesting that programs condone these trainee-industry interactions. Specialty societies, medical educators, and sponsoring institutions should reexamine how they address potential conflicts of interest in training programs.

## Notes

Supplement 1. **eTable 1. **Types of Industry Largess Received by Residents in 6 Medical Specialties, 2020 to 2021 **eTable 2.** Types of Industry Largess Received by Residents in all Medical Specialties, 2020 to 2021 **eTable 3.** Definitions of Payment Types

Supplement 2.
Data Sharing Statement
