# Supplementary material for: Industry Payments Received by Residents During Training
Source: JAMA Netw Open. 2023 Oct 16;6(10):e2337904. doi: 10.1001/jamanetworkopen.2023.37904 (PMC10580108; doi:10.1001/jamanetworkopen.2023.37904)
Supplement: Supplement 1. — eTable 1. Types of Industry Largess Received by Residents in 6 Medical Specialties, 2020 to 2021 eTable 2. Types of Industry Largess Received by Residents in all Medical Specialties, 2020 to 2021 eTable 3. Definitions of Payment Types [file jamanetwopen-e2337904-s001.pdf]

Supplemental Online Content

Hogan S, Yamazaki K, Jing Y, Trock B, Han M, Holmboe E. Industry payments received by residents during training. *JAMA Netw Open*. 2023;6(10):e2337904. doi:10.1001/jamanetworkopen.2023.37904

eTable 1. Types of Industry Largess Received by Residents in 6 Medical Specialties, 2020 to 2021
eTable 2. Types of Industry Largess Received by Residents in all Medical Specialties, 2020 to 2021
eTable 3. Definitions of Payment Types

This supplemental material has been provided by the authors to give readers additional information about their work.

© 2023 Hogan S et al. *JAMA Network Open.*

eTable 1. Types of Industry Largess Received by Residents in 6 Medical Specialties, 2020 to 2021

	Count of payments												
Specialty	Food and Beverage	Education	Travel and Lodging	Grant	Consulting Fee	Compensation for services other than consulting, including serving as faculty or as a speaker at a venue other than a continuing education program	Gift	Royalty or License	Honoraria	Entertainment	Long term medical supply or device loan	Compensation for serving as faculty or as a speaker for a medical education program	Debt forgiveness
Orthopaedic surgery	5815	1675	397	36	7	7		4				1	
Internal medicine	7293	44	11	25	1	2	17						

© 2023 Hogan S et al. *JAMA Network Open.*

**Family medicine**	**4631**	**2**	**3**	**3**	**4**	**3**	**1**				**1**		
**Surgery**	**2858**	**185**	**99**	**18**	**7**	**7**	**1**						
**Urology**	**2136**	**54**	**18**	**20**		**2**							
**Obstetrics and gynecology**	**1131**	**90**			**1**		**1**						
**total**	**23864**	**2050**	**528**	**102**	**20**	**21**	**20**	**4**	**0**	**0**	**1**	**1**	**0**
**% of total**	**89.7%**	**7.7%**	**2.0%**	**0.4%**	**0.1%**	**0.1%**	**0.1%**	**0.0%**	**0.0%**	**0.0%**	**0.0%**	**0.0%**	**0.0%**

© 2023 Hogan S et al. *JAMA Network Open.*

eTable 2. Types of Industry Largess Received by Residents in all Medical Specialties, 2020 to 2021

	**Count of payments**												
**Specialty**	**Food and Beverage**	**Education**	**Travel and Lodging**	**Grant**	**Consulting Fee**	**Compensation for services other than consulting, including serving as faculty or as a speaker at a venue other than a continuing education program**	**Gift**	**Royalty or License**	**Honoraria**	**Entertainment**	**Long term medical supply or device loan**	**Compensation for serving as faculty or as a speaker for a medical education program**	**Debt forgiveness**
Orthopaedic surgery	5815	1675	397	36	7	7		4				1	
Internal medicine	7293	44	11	25	1	2	17						
Dermatology	5286	217	1		15	10							
Family medicine	4631	2	3	3	4	3	1				1		

© 2023 Hogan S et al. *JAMA Network Open.*

Surgery	2858	185	99	18	7	7	1						
Psychiatry	2732	23				2	2						
Ophthalmology	2129	116	2		1				1				
Urology	2136	54	18	20		2							
Neurological surgery	1635	100	218	4	12	6			1	1			
Neurology	1782	12	2	7		34	2						
Obstetrics and gynecology	1131	90			1		1						
Vascular surgery - integrated	1042	104	30	29		1	1			1			
Otolaryngology - Head and Neck Surgery	657	70	87		6	1							
Emergency medicine	800	7	3	1	3	2	2						
Plastic Surgery - Integrated	700	63	7		24	3							
Thoracic surgery	490	112	188	1	1								
Anesthesiology	649	12	22	12	1		1						
Plastic surgery	618	44			2								
Allergy and immunology	621	1											
Interventional radiology - integrated	550	31	2	2		1							

© 2023 Hogan S et al. *JAMA Network Open.*

Physical medicine and rehabilitation	470	15	5	4									
Radiology-diagnostic	329	16	2	1	4								
Thoracic surgery - integrated	283	40	27										
Radiation oncology	308	3			1								
Pediatrics	247	2	1				1				1		1
Transitional year	197	2					1						
Internal medicine/Pediatrics	184												
Colon and rectal surgery	100	6	42										
Child neurology	82	1		2		2			1				
Occupational and environmental medicine	52		1										
Pathology-anatomic and clinical	43												
Public health and general preventive medicine	36												
Osteopathic neuromusculo	27												

© 2023 Hogan S et al. *JAMA Network Open.*

skeletal medicine													
Emergency medicine/Family medicine (components individually accredited)	15												
Nuclear medicine	2	7											
Internal medicine/Psychiatry (components individually accredited)	5												
Family medicine/Osteopathic neuromusculoskeletal medicine (components individually accredited)	3												
Internal med/Emer med/Critical care (components individually accredited)	2												

© 2023 Hogan S et al. *JAMA Network Open.*

Internal medicine/Emergency medicine (components individually accredited)	2												
Internal medicine/Preventive medicine (components individually accredited)	2												
Medical genetics and genomics	1												
Internal medicine/Dermatology (components individually accredited)		1											
Aerospace medicine	1												
Column total	45946	3055	1168	165	90	83	30	4	3	2	2	1	1
% of total	90.9 %	6.0%	2.3%	0.3 %	0.2%	0.2%	0.1 %	0.0%	0.0%	0.0%	0.0%	0.0%	0.0%

© 2023 Hogan S et al. *JAMA Network Open.*

eTable 3. Definitions of Payment Types

**Payment type**	**CMS Definition of Payment Type**
Compensation for services other than consulting, including serving as faculty or as a speaker at a venue other than a continuing education program	Includes payments that a company makes to physicians for speaking, training, and education engagements that are not for continuing education.
Compensation for serving as faculty or as a speaker for a medical education program	Compensation for serving as faculty or as a speaker for an accredited or certified continuing education program. Compensation for serving as faculty or as a speaker for a non-accredited and noncertified continuing education program
Consulting Fee	A payment that a company makes to a physician for advice and expertise about a medical product or treatment. Consulting fees are typically arranged with a written agreement between a company and physician based on the company's particular business needs. These payments often vary depending on the consulting physician's expertise.
Debt forgiveness	Forgiving the debt of a covered recipient, a physician owner, or the immediate family of the physician.
Education	Payments or transfers of value for classes, activities, programs, or events that involve learning or teaching a profession skill. This payment can include things like textbooks and medical journal articles.
Entertainment	Attendance at recreational, cultural, sporting or other events that would generally have a cost.
Food and Beverage	Food and beverage.

© 2023 Hogan S et al. *JAMA Network Open.*

Gift	A general category which includes anything a reporting entity provides to a covered recipient that does not fit into another Nature of Payment category.
Grant	A payment to a covered recipient to support a specific cause or activity
Honoraria	Similar to consulting fees, but generally reserved for a brief, one-time activity. Another distinction is that honoraria are generally provided for services without a set price.
Long term medical supply or device loan	The loan of supplies or a device for a total of 91 days or longer, regardless of whether the loan was 90 consecutive days
Royalty or License	Payments based on sales of products that use a physician's intellectual property.
Travel and Lodging	Any compensation for costs associated with travel, such as hotel fees, airfare, mileage, and cab fare.
**Source**: CMS Open Payments website, https://www.cms.gov/openpayments/natures-of-payment	
Accessed June 1, 2023.	

© 2023 Hogan S et al. *JAMA Network Open.*
