# Supplementary material for: Industry Payments Received by Residents During Training
Source: JAMA Netw Open. 2023 Oct 16;6(10):e2337904. doi: 10.1001/jamanetworkopen.2023.37904 (PMC10580108; doi:10.1001/jamanetworkopen.2023.37904)
Supplement: Supplement 2. — Data Sharing Statement [file jamanetwopen-e2337904-s002.pdf]

Data Sharing Statement

Hogan. Industry Payments Received by Residents During Training. *JAMA Netw Open*. Published October 16, 2023. doi:10.1001/jamanetworkopen.2023.37904

Data
Data available: Yes
Data types: Deidentified participant data
How to access data: Upon request to corresponding author.
When available: beginning date: 12-05-2024, end date: 12-30-2026

Supporting Documents
Document types: None

Additional Information
Who can access the data: Qualified researchers.
Types of analyses: Validation & replication.
Mechanisms of data availability: With signed data use agreement, IRB approval, statement of purpose.
Any additional restrictions: Some data (sex, gender) was obtained by a 3rd party. Sharing sex data is subject to the approval of the AAMC.
